# Measurement of refractive indices of tunicates’ tunics: light reflection of the transparent integuments in an ascidian *Rhopalaea* sp. and a salp *Thetys vagina*

**DOI:** 10.1186/s40851-017-0067-6

**Published:** 2017-05-30

**Authors:** Hiroshi Kakiuchida, Daisuke Sakai, Jun Nishikawa, Euichi Hirose

**Affiliations:** 1 Structural Materials Research Institute, National Institute of Advanced Industrial Science and Technology (AIST), Moriyama, Nagoya, Aichi 463-6560 Japan; 2 Department of Electrical and Electronic Engineering, Kitami Institute of Technology, Koen-cho, Kitami, Hokkaido 090-8507 Japan; 3 Department of Marine Biology, School of Marine Science and Technology, Tokai University, Orido, Shimizu, Shizuoka, 424-8610 Japan; 4 Faculty of Science, University of the Ryukyus, Nishihara, Okinawa 903-0213 Japan

**Keywords:** Tunic, Transparency, Refractive index, Refractometry, Ellipsometry, Rigorous coupled wave analysis (RCWA)

## Abstract

**Background:**

Tunic is a cellulosic, integumentary matrix found in tunicates (Subphylum Tunicata or Urochordata). The tunics of some ascidian species and pelagic tunicates, such as salps, are nearly transparent, which is useful in predator avoidance. Transparent materials can be detected visually using light reflected from their surfaces, with the different refractive indices between two media, i.e., tunic and seawater, being the measure of reflectance. A larger difference in refractive indices thus provides a larger measure of reflectance.

**Results:**

We measured the refractive indices of the transparent tunic of *Thetys vagina* (salp: Thaliacea) and *Rhopalae*a sp. (ascidian: Ascidiacea) using an Abbe refractometer and an ellipsometer to estimate the light reflection at the tunic surface and evaluate the anti-reflection effect of the nipple array structure on the tunic surface of *T. vagina*. At D-line light (λ = 589 nm), the refractive indices of the tunics were 0.002–0.004 greater than seawater in the measurements by Abbe refractometer, and 0.02–0.03 greater than seawater in the measurements by ellipsometer. The refractive indices of tunics were slightly higher than that of seawater. According to the simulation of light reflection based on rigorous coupled wave analysis (RCWA), light at a large angle of incidence will be completely reflected from a surface when its refractive indices are smaller than seawater. Therefore, the refractive index of integument is important for enabling transparent organisms to remain invisible in the water column.

**Conclusion:**

In order to minimize reflectance, the refractive index should be similar to, but never smaller than, that of the surrounding seawater. The simulation also indicated that the presence or absence of a nipple array does not cause significant difference in reflectance on the surface. The nipple array on the tunic of the diurnal salp may have another function, such as bubble repellence, other than anti-reflection.

**Electronic supplementary material:**

The online version of this article (doi:10.1186/s40851-017-0067-6) contains supplementary material, which is available to authorized users.

## Background

The subphylum Tunicata (= Urochordata) is the sister group of Vertebrata and includes three classes: Ascidiacea, which comprises only sessile species, and Thaliacea and Appendicularia, which are always pelagic. Salpida is an order of the class Thaliacea, and salps are soft-bodied animals apparently lacking effective defense organs. Instead, they are often functionally invisible in the water column due to their transparency, helping them to avoid visual predators. However, their body contours can sometimes be recognized by light reflecting from the body surface. This may indicate that reducing reflection is important for the survival of salps, especially those occurring in epipelagic layers. Some salps accomplish this by dwelling in relatively deeper (i.e., darker) regions during the day and migrating to shallower regions only at night (known as diel vertical migration) [1, 2], but some species remain at a shallow depth throughout the day [3]. Some of these diurnal species, including *Thalia rhomboides* and *Thetys vagina*, have an array of nipple-like protuberances ≤ 50 nm in height on the cuticle surface of their outer integument, or tunic [4, 5]. Similar structures are well known in other invertebrate species, such as the corneal nipple array on the surface of the compound eyes of moths, which reduces light reflection [6, 7]. Simulation of light reflection using rigorous coupled wave analysis (RCWA) has indicated that the reflection on the salp nipple array is slightly lower than that on the flat surface at high incidence angles (*θ* > 80°), suggesting an anti-reflection function of the surface nanostructures [5].

Light reflection occurs at the boundary between two optical media with different refractive indices, but the refractive index of the tunic structures in tunicates is not known. Since the water content of salps is 95% or higher [8], the refractive index should not be considerably different from that of seawater. In the simulation described above, the refractive index of the tunic was assumed to be 0.1 larger than that of seawater, but a tested value rather than an assumption would be essential for producing an accurate simulation of salp light reflectance.

Among the metazoans, the ability of cellulose synthesis is exclusively found in the members of the subphylum Tunicata (or Urochordata) in which epidermal cells secrete cellulose microfibrils to form the tunic in tunicates, including salps [4], except for appendicularians, which form a cellulosic feeding-apparatus called a “house” [9]. Consequently, ascidians and thaliaceans have very unique integument, i.e., tunic, that contains cellulosic matrix and free cells outside the epidermis [10–12]. Although hardness, cellulose content, and water content vary among tunicate species [13–15], salp tunic is usually gelatinous and transparent. The refractive indices of these cellulosic matrices have not been reported to date.

In the present study, we measured the refractive indices of the transparent tunic of a salp, *Thetys vagina*, and an ascidian, *Rhopalaea* sp., using an Abbe refractometer and an ellipsometer. For *T. vagina*, only frozen specimens were examined; for *Rhopalaea* sp., both fresh and frozen materials were compared. The tunics of both species appeared completely transparent, and they are free from debris and epibionts that alter the optical properties in natural condition. We also measured the absorption spectra of the transparent tunic specimens to consider the tunic function as protection against ultraviolet (UV) radiation in shallow waters. Using this information, we proposed a surface structure model of *T. vagina* tunic based on ultrastructural observation. Simulation of light reflectance on the tunic surface with or without nipple array structures was carried out based on the estimated refractive indices to elucidate whether the array provides effective reduction of light reflection.

## Methods

### Animals

**Fig. 1:**
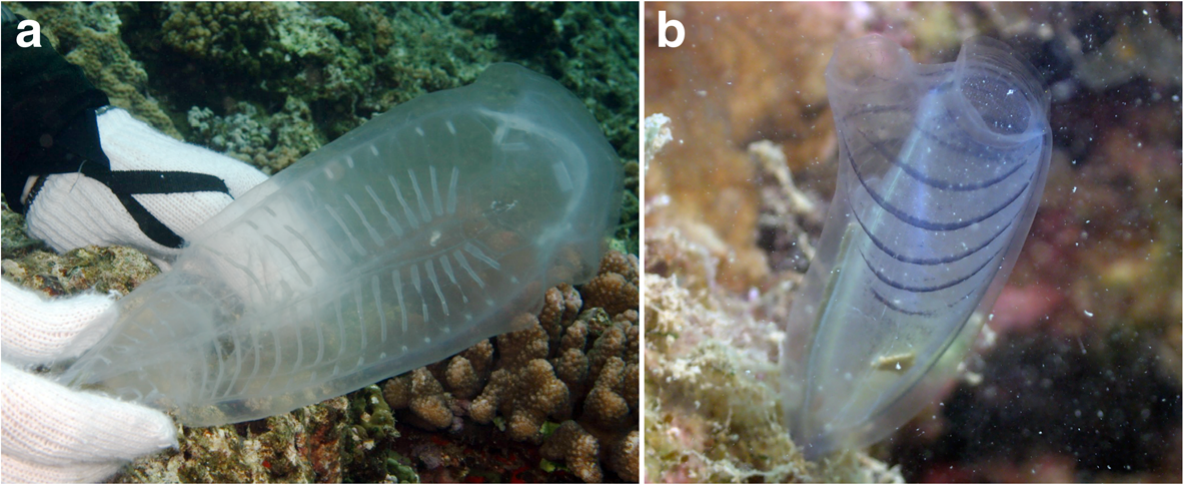
**a** *Thetys vagina* collected near Cape Zanpa (photo: James-Davis Reimer). **b** *Rhopalaea* sp. found in Kin Bay (photo: Ryosuke Uozumi)

A solitary form of the salp *Thetys vagina* (body length = 16 cm) was collected at Sunakohama port, Okirai Bay, Iwate, Japan using a scoop net. A second specimen of the same species was collected using SCUBA at 35 m depth off Cape Zanpa, Okinawajima Island, Okinawa, Japan (Fig. 1a). These specimens were transferred to the laboratory and stored at −20 °C without any cryoprotectants. The specimens were then thawed at room temperature, and 2 × 2 cm sheets of tunic of 3 mm depth were cut from the middle part of the bodies with a razor. The attached mantle tissue below the tunic was removed using forceps. The resulting tunic sample was transparent and often had a warty surface. A flat area was selected for measurement.

Fifteen individuals of the ascidian *Rhopalaea* sp. (ca. 10 cm in length) were collected using SCUBA in Kin Bay (Okinawajima Island, Okinawa, Japan) at approximately 3 m depth (Fig. 1b). Further specimens for electron microscopy were collected off Sunabe (Okinawajima Island, Okinawa, Japan) at approximately 15 m depth. These were reared in seawater or artificial seawater (Marine Art SF-1: Osakayakken Co., Ltd., Minou, Osaka, Japan) for 1–2 days prior to use. Sheets of tunic 2 × 2 cm and 1 mm thick were cut from fresh specimens and the mantle tissue below the tunic was removed with forceps, yielding clear tunic material with a flat surface. Four of these samples were frozen at −20 °C and thawed at room temperature before the measurement.

All procedures conducted in this study comply with the local and institutional ethical guidelines.

### Absorption spectra of tunics

The tunics of *T. vagina* (from a previously frozen specimen) and *Rhopalaea* sp. (from a fresh specimen) were each attached to the wall of a quartz cell, and the 280–800 nm absorption spectra were recorded at 2-nm intervals with a U-4100 spectrophotometer (Hitachi, Tokyo, Japan). As reference for a tunic containing UV-absorbing substances, we used the absorption spectra of *Diplosoma virens*, a colonial ascidian containing symbiotic cyanobacteria measured in a previous study by our group [16].

### Refractometry with an Abbe refractometer

Refractive indices of the tunic specimens were measured using an Abbe refractometer (NAR-1 T SOLID: Atago Co., Ltd., Tokyo, Japan) at 24.5–25.0 °C with LED light approximating to the wavelength of D-line (ca. 589 nm). This wavelength originated from the emission spectrum of sodium and has been conventionally used for the measurement of refractive indices as a standard. According to the manufacturer’s specifications, the measurement accuracy of this refractometer is ±0.0002 (nD). The outer surface of the tunic, i.e., cuticular side, faced the prism of the refractometer. Refractive indices (nD) of seawater and artificial seawater were also measured at 25 °C, and found to be 1.3392–1.3394. The measured values of these tunic specimens and seawater were compared with Steel-Dwass test using Satcel4 (OMS Publishing Inc., Tokorozawa, Japan).

### Ellipsometry

We examined the tunics of *T. vagina* (previously frozen specimens) and *Rhopalaea* sp. (fresh specimens) by ellipsometric measurement. The wavelength dispersion of refractive index was determined in the range between 380 and 1700 nm, using a spectroscopic ellipsometer (M2000, J. A. Woolam Co. Inc., Lincoln, NE, USA). Focusing probes were installed in this ellipsometric system to find a flat surface of the specimens placed on a flat plate. The measurements were carried out at different angles of incidence from 50 to 80° at 5° intervals. The thickness of these samples was large enough to avoid stray light from backside reflection through the transparent body of the specimens. The ellipsometric parameters *Ψ* and *Δ* were measured and were analyzed assuming Cauchy expansion [17].

### Electron microscopy

Tunic sheets of the thawed *T. vagina* tunic specimen and the fresh *Rhopalaea* sp. tunic specimen were fixed with 2.5% glutaraldehyde (16320-P; Electron Microscopy Sciences, Hatfield, PA, USA) in a 0.45 M sucrose and 0.1 M sodium cacodylate (S008; TAAB Laboratories, Berks, England) buffer (pH 7.5) at room temperature and stored at 4 °C. The specimens were briefly rinsed with the same buffer, post-fixed with 1% osmium tetroxide (124505; Merck, Darmstadt, Germany) in a 0.1 M cacodylate buffer (pH 7.5) at 4 °C for 1.5 h, and dehydrated through an ethanol series. Specimens were then embedded in an epoxy resin (T024; TAAB Laboratories) for transmission electron microscopy (TEM). Sections of 0.1 μm thick were stained with uranyl acetate and lead citrate and examined under TEM (JEM-1011; JEOL, Tokyo, Japan) at 80 kV. The heights and intervals of the cuticular protuberances were measured from electron micrographs.

### Simulation of light reflection with and without a nipple array

The light reflection was calculated at the border between the medium (seawater) and matrix (tunic) with or without the nipple array with rigorous coupled wave analysis (RCWA) using DiffractMOD3.2 software (RSoft Design Group, Inc., Ossining, NY, USA). The differences between refractive indices between the tunic and seawater were assumed to be −0.04, −0.02, 0.02, and 0.04, according to estimates of refractive indices measured with the refractometer and the ellipsometer. Polarized light was used for this simulation, i.e., transverse electric wave (TE wave) and transverse magnetic wave (TM wave). Based on the electron microscope observations, the modeling of the nipple array of *T. vagina* was assumed to be hemispheres (60 nm in radius) in which the distance between adjacent nipples’ apices was 140 nm. Parameters for the simulation included the wavelength of light (*λ*: 633 nm) and the angle of incidence of light (*θ*: 0–90°).

## Results

### Absorption spectra of the tunics

**Fig. 2:**
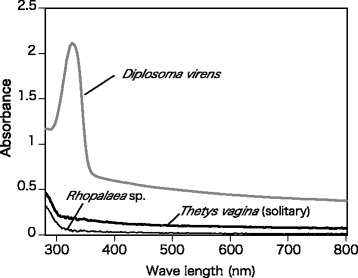
Absorption spectra of the tunic of *Thetys vagina* (*black thick line*), *Rhopalaea* sp. (*black thin line*), and *Diplosoma virens* (*grey line*). The spectra of *T. vagina* and *Rhopalaea* sp. have no prominent peaks, whereas the spectrum of *D. virens* has an absorption peak due to UV-absorbing substances

In *T. vagina* and *Rhopalaea* sp., the absorption spectra of the tunic showed no specific absorption peaks in the range of visible light (400–760 nm), UV-A (325–400 nm), or UV-B (280–315 nm) (Fig. 2). In *D. virens* tunic containing UV-absorbing substances, the absorption spectra had a prominent absorption peak at around 320 nm (Fig. 2).

### Measurement by an Abbe refractometer

When tunic samples of *Rhopalaea* sp. were set in the refractometer, upper and lower boundaries of the refractive index were observed. The refractive indices of the upper boundary ranged from 1.3384 to 1.3408 (*n* = 15, mean = 1.3394, SD = 0.0006), similar to those measured in seawater (1.3392–1.3394). The refractive indices of the lower boundaries were approximately 1.3435, yielding a difference 0.004 between of the upper and lower boundary (Table 1). When *T. vagina* samples were measured, only one boundary was observed. This was comparable to those of the lower boundary in the measurement of *Rhopalaea* sp., and was measured at approximately 1.3416, or 0.002, which differed from the refractive index of seawater (1.3393) (Table 1). As shown in Additional file 1, there were no significant differences among the measured values of the tunic specimens (i.e., fresh and frozen specimens of *Rhopalaea* sp. and frozen specimens of *T. vagina*), whereas these values were significantly larger than the values of the upper boundary, i.e., seawater (Steel-Dwass test: *P* < 0.01 for seawater– fresh *Rhopalaea* sp., *P* < 0.05 for seawater – frozen *Rhopalaea* sp. and seawater – frozen *T. vagina*).

**Table 1:** Refractive indices of the tunic measured by an Abbe refractometer

Specimens	Number	Refractive indices: Average (± SD)		
		Upper boundary	Lower boundary	Difference between the boundaries
*Rhopalaea* sp. (fresh)	11	1.3393 (±0.0006)	1.3435 (±0.0017)	0.0042 (±0.0016)
*Rhopalaea* sp. (frozen–thawed)	4	1.3396 (±0.0002)	1.3434 (±0.0003)	0.0038 (±0.0002)
*Thetys vagina* (frozen–thawed)	4	Not available	1.3416 (±0.0011)	0.0023 (±0.0011)^a^

^a^Difference of refractive indices between the boundary and the seawater (nD = 1.3393)

### Measurement by an ellipsometer

**Fig. 3:**
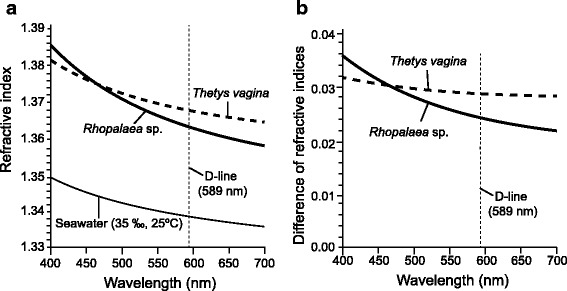
Refractive indices of tunics and seawater (**a**), and the difference between the two, in the visual light range (400–700 nm) (**b**). Refractive indices of the tunic of *Thetys vagina* (previously frozen specimen) and *Rhopalaea* sp. (fresh specimen) were calculated from ellipsometric parameters *Ψ* and *Δ* (Additional files 2 and 3). The *curve* for seawater is based on the equation of Quan and Fry [22] that is valid from 400 to 700 nm. The *vertical dotted line* indicates the wavelength of D-line that was used for the measurement with the Abbe refractometer

Ellipsometric parameters *Ψ* and ***Δ*** of *T. vagina* tunic specimens (previously frozen) and *Rhopalaea* sp. (fresh specimens) were obtained at angles of incidence from 50 to 80° at 5° intervals (Additional files 2 and 3). Refractive index (*n*) was calculated with the constraint of Cauchy expansion, and the values were determined as expressed by the equations below.

$$ Rhopalaea\;\mathrm{s}\mathrm{p}.:\kern0.48em  n=\kern0.5em \left(1.3447\kern0.5em \pm \kern0.5em 0.0012\right)\kern0.5em +\kern0.5em \left(6525.1 \pm 388.0\right)/{\uplambda}^2 $$

$$ Thetys\; vagina:\kern0.36em  n=\left(1.3563\pm 0.0021\right)+\left(4013.4\pm 588.0\right)/{\uplambda}^2 $$

where the coefficient errors were obtained by the fitting calculations, and are plotted as a function of wavelength (*λ*) in Fig. 3a. The optical structure of the surface was modeled as a homogeneous bulk for the calculation, and consequently the simplest model was most likely valid because the mean squared error was < 10 in the fitting calculations between the measured and theoretical data in the ellipsometric parameters. At a wavelength of 589 nm (D-line), the refractive indices were estimated as 1.364 in *Rhopalaea* sp. (fresh specimen) and 1.368 in *T. vagina* tunic (previously frozen specimen).

### Fine structures of tunic surface

**Fig. 4:**
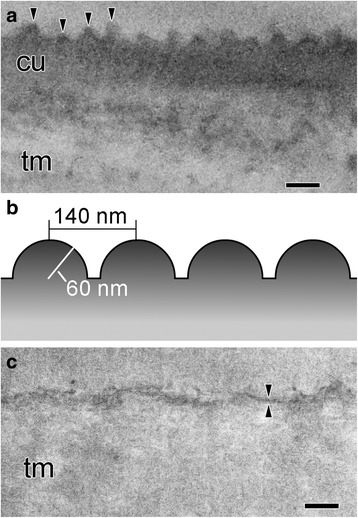
Fine surface structure of *Thetys vagina* tunic (**a** TEM; **b** model for the simulation of light reflectance) and *Rhopalaea* sp. tunic (**c** TEM). *Arrowheads* in **a** indicate cuticular protuberances, and facing *arrowheads* in **c** indicate the cuticular layer. cu, cuticular layer; tm, tunic matrix. Scale bars in **a** and **c** indicate 0.2 μm

In *T. vagina*, the tunic matrix consisting of fuzzy materials was entirely covered with the tunic cuticle, an electron-dense layer approximately 0.2 μm thick (Fig. 4a). There was an array of cone-shaped or hemispherical protuberances on the cuticle surface (Fig. 4a). The height of these protuberances was 0.057 ± 0.011 μm (average ± SD, *n* = 18) and the distance between the apices of adjacent protuberances was 0.138 ± 0.045 μm (average ± SD, *n* = 17). Based on this observation, we hypothesize the surface structure model of the *T. vagina* tunic as hemispheres (60 nm in radius) arranged in 140-nm intervals for the simulation below (Fig. 4b). The tunic of *Rhopalaea* sp. had a very thin cuticular layer (<40 nm thick) that was composed of electron-dense, fibrous materials (Fig. 4c). The cuticular layer was often frayed, suggesting that the layer was not structurally firm. Therefore, the cuticular surface was not always flat, likely due to damage, but there were no regular, repetitive structures, such as nipple arrays, on the cuticular surface.

### Simulation of light reflection on the tunic surface with and without a nipple array

**Fig. 5:**
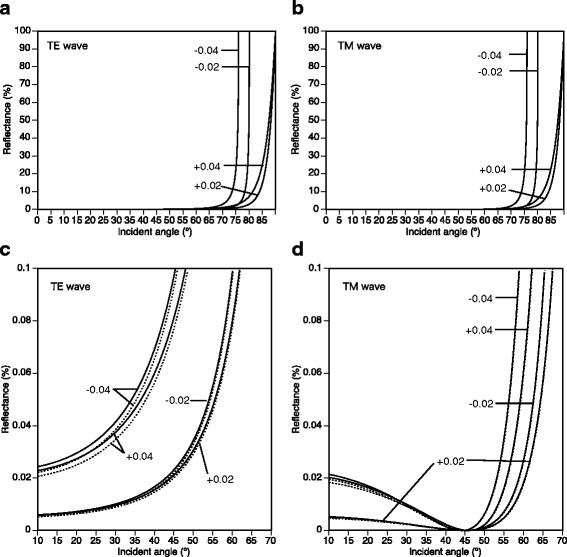
Reflectance (%) of 633 nm light [TE wave (**a**, **c**) and TM wave (**b**, **d**)] on the flat surface (*solid line*) and on the model of *Thetys vagina* tunic surface (*dotted line*). The difference in refractive index between the tunic and medium (seawater) were assumed to be −0.04, −0.02, 0.02, and 0.04. **c** and **d** show enlargements of **a** and **b**, respectively

The ellipsometric measurement indicated that the refractive indices of the tunic are quite similar to seawater, but we assumed that rigorous values could not be determined probably because of the structural unevenness of the tunics. Here, we used four sets of the refractive indices of the tunic, i.e., ± 0.02 and 0.04 of the refractive index of seawater, to simulate the light reflection on the *T. vagina* tunic. The range of these estimates are somewhat larger than the measurement by refractometry (0.002–0.004 at D-line, Table 1) and ellipsometry (0.02–0.03 at D-line, Fig. 3b), in order to account for potential errors in measurement and fluctuations of natural conditions such as temperature and salinity. In the RCWA analysis, the reflectance against incident angle resulted in J-shaped curves (Fig. 5). The reflectance curves were nearly identical, irrespective of the presence or absence of the nipple array in the all sets of the refractive indices, while the curves were quite different among the different refractive indices (Fig. 5a, b). The reflectance curves for TE wave were very similar to those of the same refractive index for TM wave. When the refractive indices were smaller than seawater (i.e., −0.02 and −0.04), light was completely reflected at angles larger than 76° and 80.1°, respectively. In contrast, when the refractive indices were larger than seawater (i.e., 0.02 and 0.04), complete reflection did not occur at angles below 90°, with 95% reflection occurring at about 89.8°. In the enlargement of the reflection curves (Fig. 5c, d), reflectance on the nipple array was slightly smaller than reflectance on the flat surface. In reflection of TM wave, the reflectance curve had a minimum at around 45° as a result of Brewster’s angle.

## Discussion

### Transparency of the tunics

The apparent transparency of the tunic in *T. vagina* and *Rhopalaea* sp. is consistent with the absorption spectra of these species that have no prominent absorption peaks in the range of 280–800 nm (Fig. 2). The tunic of another diurnal salp, *Thalia rhomboides*, has a similar absorption spectrum lacking prominent peaks [5]. These tunics are therefore transparent to many potential predators, including fish with eyesight sensitive to UV as well as visible light [18]. However, this also indicates that the tunic of these species does not protect inner tissues from UV radiation. As *Thetys vagina* and *Thalia rhomboides* are diurnal salps and *Rhopalaea* sp. is sessile in shallow coral reefs, where there is considerable UV penetration [19], this exposure could be harmful. This may explain the green pigmentation in the alimentary tract of *T. vagina.*

### Estimation of refractive indices of the tunics

In the measurement of *Rhopalaea* sp. using an Abbe refractometer, two boundaries were observed, with the refractive indices of the upper boundaries almost equal to those of the seawater or artificial seawater in which *Rhopalaea* sp. individuals were incubated. Therefore, we assumed that the upper boundary indicates the refractive index of liquid between the tunic and the prism, with this liquid possibly composed of seawater and exudate from the tunic. When the refractive index of the lower boundary is assumed to be that of the tunic surface, the difference of the refractive indices between seawater and tunic is approximately 0.004 (D-line, λ = 589 nm). Freezing did not cause considerable change of the refractive index of the tunic in *Rhopalaea* sp. In the measurement of *T. vagina* tunic, the refractive index of the boundary was approximately 1.3416. When this value is assumed to represent the refractive index of the tunic, the difference of the refractive indices between seawater and tunic is approximately 0.002 (D-line, λ = 589 nm).

At the 589 nm wavelength, refractive indices obtained from the ellipsometric analysis were 1.364 in *Rhopalaea* sp. and 1.368 in *T. vagina*. These values are approximately 0.03 larger than the values measured by the Abbe refractometer. We posit that these deviations are the result of evaporation, as samples were exposed to air for approximately one hour during the ellipsometric measurement, while samples were tightly sandwiched between prism and cover during Abbe refractometer measurement. The refractive index of cellulose, the main component of the tunic, is larger than seawater, e.g., 1.4701 at 589 nm light [20], and thus it is reasonable to conclude that the refractive index of the specimen increases during surface evaporation. In some cases, the ellipsometric measurements were unsuccessful when specimens became overly dry. This highlights the technical challenge of examining biological specimens containing water in a cellulose matrix, as water content depends on ambient conditions. The uneven surface of these specimens in comparison to synthetic materials presents additional problems, although flat areas of the specimens were measured as much as possible.

The measured values of the refractive index of tunic samples were 0.002–0.004 larger than seawater in the measurements by Abbe refractometer and 0.02–0.03 larger than seawater in the measurements by ellipsometer at D-line (λ = 589 nm), which begs the question as to which is the more representative value. As discussed above, the present measurements included some assumptions, and the measured values should be regarded as estimations. Both of the measured values of the two methods were slightly larger than the refractive index of seawater. Considering the possible sources of measurement errors, the true refractive indices of the tunics likely lie between the values measured by the two methods described. That is, Abbe refractometer measurements would underestimate refractive index, owing to the seawater between the tunic surface and the prism, while ellipsometer measurements would overestimate refractive index due to the evaporation of water and the increase of salt concentration in the specimens during the measurement. Accordingly, the refractive indices of the tunics are estimated to be slightly greater (<0.03) than seawater at D-line (λ = 589 nm).

In the *Rhopalaea* sp. tunic, the refractive indices measured by an Abbe refractometer were almost the same between the fresh specimens and the previously frozen specimens, indicating that the freeze-thaw cycle had no effect. However, we could not verify the effect of the freezing and thawing in the ellipsometric measurement.

### Reflectance of the tunic and nipple arrays

As described, the difference in refractive index between tunic and seawater is estimated as 0.03 or less. On the other hand, the refractive index of seawater in situ is variable to some extent, depending on some factors such as salinity and temperature. Therefore, the difference of the refractive index between tunic and seawater might occasionally be larger than the present estimations. In our simulations of light reflection on the tunic, the differences in refractive indices between the tunic and seawater were assumed to be −0.04, −0.02, 0.02, and 0.04. We believe that this range of assumptions is large enough to cover measurement errors and the potential change of refractive index of seawater in situ. As shown in Fig. 5, anti-reflection effect of nipple array, so called moss-eye effect, is larger when the difference of refractive indices is larger. In the case of the moth eye, the difference is 0.5 or larger; the refractive index of an insect’s chitinous tissue is > 1.54 [21] and that of air is 1.000 [20] at 589 nm. However, the difference of refractive indices between the tunics and seawater appears so small (<0.03) that the nipple array on the salp tunic rarely causes a significant anti-reflection effect.

The difference in refractive index between tunics (based on ellipsometry) and seawater at 35‰ and 25 °C (based on the equation of Quan and Fry [22]) is larger in shorter wavelength light (Fig. 3a), as is common for the refractive indices of transparent media [23]. Owing to the difference in curve shape, the difference in refractive index between seawater and tunics is larger in shorter wavelength light, particularly between seawater and the *Rhopalaea* tunic (Fig. 3b). Since blue light (470–480 nm) penetrates seawater to greater depths than other visible light wavelengths in marine environments, it has been proposed that many marine animals have visual acuity in this range [24]. The present results indicate that shorter wavelength vision would be more advantageous for predators of salps to see transparent tunic with light reflection in the water column.

## Conclusion

Measurements using both Abbe refractometer and ellipsometer showed that the refractive indices of tunics were slightly greater than that of seawater. This is the first report of measurements of refractive indices in tunicate tunics. The present simulation suggests that the refractive index of tunic should not be smaller than the seawater, because total reflection would make the salps and ascidians more conspicuous in the euphotic zone of the water column. We have not previously observed total reflection from the tunic surface in the field or in the laboratory with various angles of illumination. Accordingly, the refractive index of the tunic is hypothesized to be slightly greater than the refractive index of seawater. This is consistent with both refractometer and ellipsometer measurements. Furthermore, the presence or absence of a nipple array does not cause significant difference in reflectance on the surface (Fig. 5).

This raises the question as to the reason for the presence of a nipple array in *T. vagina* tunic. The anti-reflection function of the nipple array is very small when the refractive index of the material (tunic) is close to that of the surrounding medium (seawater). However, even this small difference in reflectance may translate to improved survival. On the other hand, the nipple array on the tunic surface may serve additional and/or alternative functions. Indeed, even if their function is at now undetermined, recent studies demonstrated that a synthetic, hydrophilic nipple array repels bubbles [25] and suppresses cell spreading [26]. These may be important functions for diurnal salps in controlling buoyancy and defending against parasites.

## Additional files

Additional file 1: Refractive indices of the tunic specimens and seawater measured by an Abbe refractometer. The refractive indices for seawater are the measured values of the upper boundary in the measurement of *Rhopalaea* specimens. Statistical significance was examined by Steel-Dwass test for nonparametric, multiple comparison. (DOCX 190 kb)

Additional file 2: Ellipsometric parameter *Ψ* (A) and ***Δ*** (B) of the tunic of *T. vagina* (previously frozen specimens). (DOCX 305 kb)

Additional file 3: Ellipsometric parameter *Ψ* (A) and ***Δ*** (B) of the tunic of *Rhopalaea* sp. (fresh specimen). (DOCX 359 kb)

## Abbreviations

RCWA: Rigorous coupled wave analysis
TE wave: Transverse electric wave
TM wave: Transverse magnetic wave
UV: Ultraviolet
