# Supplementary material for: Measurement of refractive indices of tunicates’ tunics: light reflection of the transparent integuments in an ascidian Rhopalaea sp. and a salp Thetys vagina
Source: Zoological Lett. 2017 May 30;3:7. doi: 10.1186/s40851-017-0067-6 (PMC5448145; doi:10.1186/s40851-017-0067-6)

**Additional file 1.** Refractive indices of the tunic specimens and seawater measured by an Abbe refractometer. The refractive indices for seawater are the measured values of the upper boundary in the measurement of *Rhopalaea* specimens. Statistical significance was examined by Steel-Dwass test for nonparametric, multiple comparison.


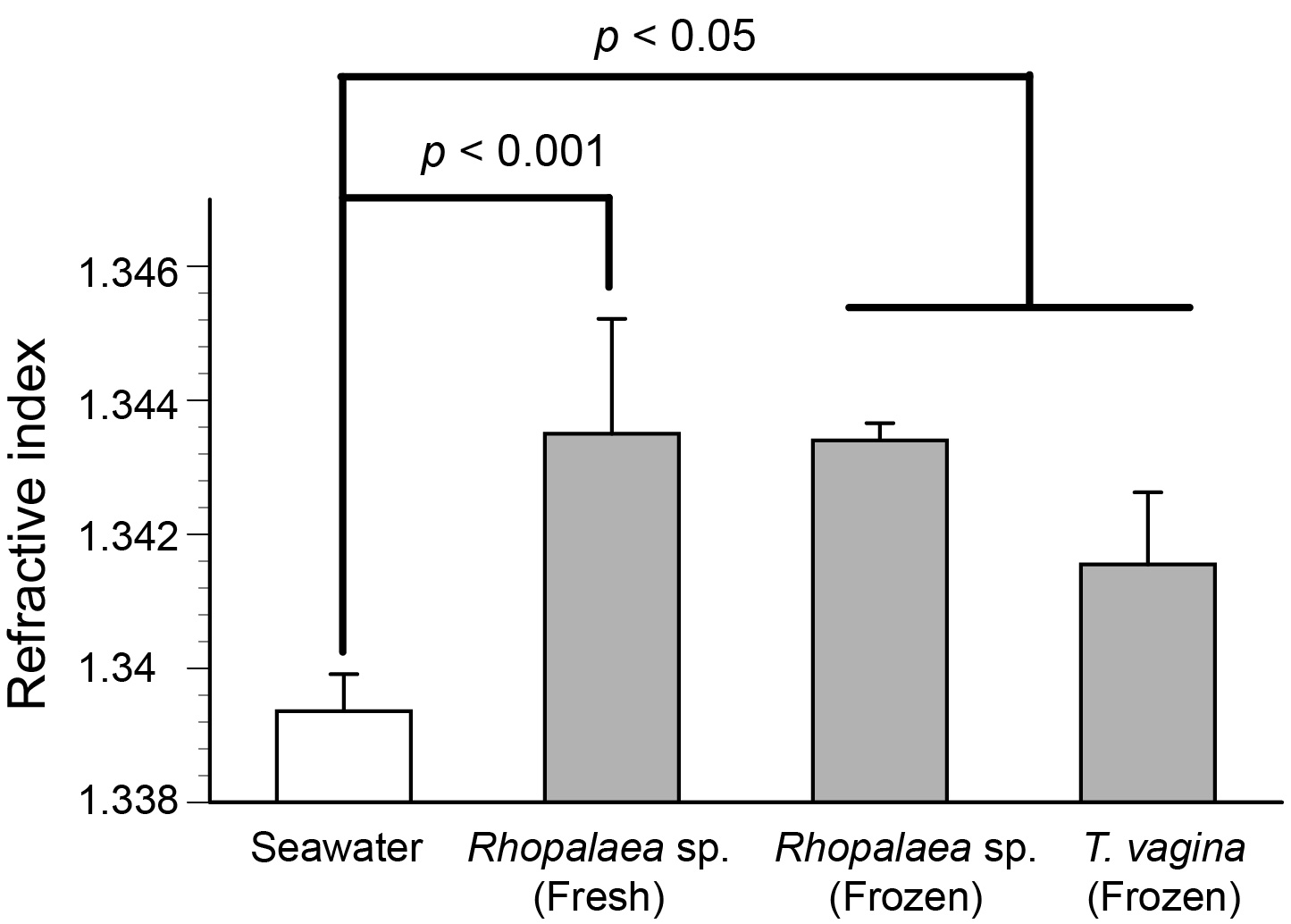

Supplement: Supplementary file 1 — Refractive indices of the tunic specimens and seawater measured by an Abbe refractometer. The refractive indices for seawater are the measured values of the upper boundary in the measurement of Rhopalaea specimens. Statistical significance was examined by Steel-Dwass test for nonparametric, multiple comparison. (DOCX 190 kb) [file 40851_2017_67_MOESM1_ESM.docx]
