# Supplementary material for: Measurement of refractive indices of tunicates’ tunics: light reflection of the transparent integuments in an ascidian Rhopalaea sp. and a salp Thetys vagina
Source: Zoological Lett. 2017 May 30;3:7. doi: 10.1186/s40851-017-0067-6 (PMC5448145; doi:10.1186/s40851-017-0067-6)
Supplement: Supplementary file 3 — Ellipsometric parameter Ψ (A) and Δ (B) of the tunic of Rhopalaea sp. (fresh specimen). (DOCX 359 kb) [file 40851_2017_67_MOESM3_ESM.docx]

**Additional file 3.** Ellipsometric parameter *Ψ* (A) and ***Δ*** (B) of the tunic of *Rhopalaea* sp. (fresh specimen).

**A**

**B**
